# Neferine inhibits angiotensin II-induced rat aortic smooth muscle cell proliferation predominantly by downregulating fractalkine gene expression

**DOI:** 10.3892/etm.2014.1952

**Published:** 2014-09-09

**Authors:** LULU ZHENG, YONGWEN CAO, SHAO LIU, ZHENYU PENG, SAIDAN ZHANG

**Affiliations:** 1 Department of Cardiology, Xiangya Hospital, Central South University, Changsha, Hunan 410008, P.R. China; 2 Department of Pharmacy, Xiangya Hospital, Central South University, Changsha, Hunan 410008, P.R. China; 3 Department of Emergency, Second Xiangya Hospital, Central South University, Changsha, Hunan 410011, P.R. China

**Keywords:** neferine, angiotensin II, fractalkine, rat aortic smooth muscle cells, proliferation

## Abstract

Neferine inhibits the angiotensin II (AngII)-induced proliferation of vascular smooth muscle cells (SMCs), but the underlying mechanism is unclear. The aim of this study was to explore the mechanism underlying the effect of neferine on the proliferation of vascular SMCs. Rat aortic SMCs (RASMCs) were used and fractalkine (Fkn) gene expression was measured by quantitative polymerase chain reaction and western blot analysis. The proliferation of RASMCs was analyzed by MTT assay and flow cytometry. It was revealed that AngII induced Fkn expression in a dose- and time-dependent manner. Fkn-knockdown with small interfering RNA attenuated the AngII-induced RASMC proliferation. Furthermore, neferine inhibited Fkn expression and attenuated the AngII-induced RASMC proliferation. These findings suggest that the Fkn gene may play an important role in AngII-induced RASMC proliferation and that neferine acts to attenuate AngII-induced RASMC proliferation by inhibiting Fkn expression.

## Introduction

The proliferation of smooth muscle cells (SMCs) plays a pivotal role in cardiovascular diseases (1–3). However, the underlying mechanism remains unclear. The renin-angiotensin-aldosterone system (RAAS) is involved in endothelial dysfunction, vascular remodeling, oxidative stress, proinflammatory cytokine production and adhesion molecule synthesis in the vascular wall (4). As an important part of the RAAS, angiotensin II (AngII) is able to stimulate the growth and proliferation of vascular SMCs by binding to the AngII receptor Type 1. AngII activates downstream signaling molecules, such as protein kinase C and mitogen-activated protein kinase (MAPK), and a number of extracellular signaling molecules, including extracellular signal-regulated kinase (ERK) 1/2, p38MAPK, c-Jun N-terminal kinase and ERK5, to carry out its functions (5–8).

Chemokines are low molecular-mass cytokines that, based on the spacing of N-terminus cysteine residues, are classified into the C, CC, CXC and CX3C families; fractalkine (Fkn; CX3CL1) is the only member of the CX3C family identified to date (9). Studies have shown that Fkn is involved in various inflammatory diseases, including cardiovascular diseases (10–12). Sullivan *et al* (13) found that the expression of Fkn was markedly increased in the mesenteric arteries of spontaneously hypertensive rats (SHRs) and was notably higher in female SHRs. Studies have also demonstrated that Fkn can induce SMC proliferation, which is mediated by nuclear factor κB-dependent inflammatory signals (14,15). Recently, Rius *et al* (16) found that by stimulating endothelial cells with 1 μmol/l AngII, Fkn expression was upregulated in the cells. These findings highlight the significance of Fkn in the pathogenesis of hypertension and suggest it may play a role in vascular remodeling.

Neferine is extracted from the herb *Nelumbo nucifera* (Gaertn.), an ingredient in Traditional Chinese Medicine. Studies have shown that *N. nucifera* has multiple biological activities, including attenuating bleomycin-induced pulmonary fibrosis (17), enhancing insulin sensitivity in insulin-resistant rats (18) and exerting an antioxidant effect against isoproterenol-induced oxidative stress (19). Previous studies have found that *N. nucifera* leaf extract and neferine can inhibit the proliferation of vascular SMCs; however the underlying mechanism has yet to be elucidated (20,21).

Based on these previous findings on the role of Fkn in the pathogenesis of various cardiovascular diseases, on the proliferative effects of AngII on vascular SMCs and on the effects of *N. nucifera* and neferine, we hypothesized that Fkn could be involved in the AngII-induced proliferation of vascular SMCs, and that the anti-proliferative effects of neferine on the cells could be fundamentally associated with Fkn and AngII. The aim of the present study was to explore the mechanisms underlying the effects of AngII and neferine on rat aortic smooth muscle cells (RASMCs).

## Materials and methods

### Materials

The RASMC line was obtained from the American Type Culture Collection (Manassas, VA, USA). The AngII, propidium iodide (PI) solution, bicinchoninic acid (BCA) protein assay and RNase A were purchased from Sigma (St. Louis, MO, USA). Goat polyclonal anti-Fkn antibody and mouse monoclonal anti-β-actin antibody were obtained from Santa Cruz Biotechnology, Inc. (Santa Cruz, CA, USA). Neferine, with a purity of 98.6%, was extracted from the seed embryo of *N. nucifera* using a preparative high-speed counter-current chromatography method by the Department of Pharmacy, Xiangya Hospital (Central South University, Changsha, China) (22).

### Cell culture and experimental design

The cells were grown in Dulbecco’s Modified Eagle’s Medium (DMEM; Hyclone, Logan, UT, USA) supplemented with 10% fetal bovine serum (FBS; Hyclone) at 37°C in a humidified atmosphere of 5% CO_2_. Cells between four and seven passages were used for all the experiments in this study. RASMCs were seeded on 96-well plates (0.2–1.0×10^4^ cells/well) or six-well culture plates (1×10^5^ cells/well) and cultured for 24 h, prior to being starved for 24 h in DMEM containing 0.1% FBS. Different concentrations of AngII (1×10^−6^, 1×10^−7^ and 1×10^−8^ M) were then added and the cells were cultured for a certain time-period according to the experimental design (6, 12 or 24 h) to evaluate the effects of AngII on RASMC proliferation and Fkn expression. To examine the effect and determine a suitable concentration of neferine on the proliferation of RASMCs, the cells were divided into four groups: Control and neferine treatment with three different concentrations of neferine (1, 5 and 10 μmol/l obtained by dilution with DMEM). The cells were then cultured for another 24 h prior to an MTT assay being performed. In order to conduct all the other experiments in the neferine study, the cells that had reached synchronization were divided into three groups: Control, AngII (1×10^−6^ M) and neferine plus AngII (neferine at 5 μmol/l, preculture for 1 h and the subsequent addition of AngII at 1×10^−6^ M). The cells were cultured for a further 24 h prior to undergoing analyses, including MTT assay, flow cytometry, western blotting and the quantitative polymerase chain reaction (qPCR). For RNA interference and cell transfection, the cells were divided into four groups: Control, AngII (1×10^−6^ M), control small interfering (si)RNA (following transfection with control siRNA, AngII at 1×10^−6^ M was added) and Fkn siRNA plus AngII (following transfection with Fkn siRNA, AngII at 1×10^−6^ M was added). The cells were subsequently cultured for 24 h for the MTT assay and flow cytometry. All the experiments were performed in triplicate. The study was approved by the Ethics Committee of Xiangya Hospital.

### MTT assay

Subsequent to finishing the cell culture according to the experimental design, 20 μl MTT (5 mg/ml) solution was added and incubated for 4 h. The supernatants of the cell culture were then removed and 150 μl dimethylsulfoxide was added to each well. A multi-well plate reader (Model Stat-Fax-2100, Awareness Technology Inc., Palm City, FL, USA) was subsequently used to determine the optical density (OD). The absorbance of samples was measured at 570 nm (OD_570_), and the absorbance at 690 nm was used as a reference. The background absorbance of the medium was also subtracted. Each assay was repeated in triplicate, and the mean for each experiment was calculated.

### Flow cytometric determination of cell cycles

At the end of cell culturing, the cells were trypsinized and washed twice with cold phosphate-buffered saline (PBS). The cells were then fixed with ice-cold 70% ethanol at 4°C overnight. The cells were subsequently centrifuged to remove fixative solution and washed with PBS twice, and then stained with 50 μg/ml PI solution and RNAse for 30 min in the dark. A total of ≥10,000 cells were analyzed. The percentages of cells in different phases of the cell cycle were measured with a fluorescence-activated cell sorting flow cytometer (Cytomics™ FC 500; Beckman Coulter, Miami, FL, USA) and analyzed with CXP software (Beckman Coulter). The percentage of cells in the S and G_2_/M phases was used as the indicator for cell proliferation.

### RNA interference and cell transfection

To confirm the role of Fkn in the AngII-induced proliferation of RASMCs, Fkn siRNA was developed to silence Fkn gene expression, as Fkn expression has been found in RASMCs under certain stimuli (23,24). The Fkn siRNA (sense strand, 5′-GCU GUGGUAGUAAUUCAUAdTdT-3′ and antisense strand, 3′-dTdTCGACACCAUCAUUAAGUAU-5′) was synthesized by RayBiotech (Guangzhou, China) and was transfected into RASMCs using Lipofectamine^®^ 2000 (Invitrogen Life Technologies, Carlsbad, CA, USA) according to the manufacturer’s instructions. Transfection efficiency was determined by qPCR and western blot analysis.

### Western blot analysis

The cells were harvested at the end of culture and the proteins were obtained by use of radioimmunoprecipitation assay buffer containing 1 mM phenylmethylsulfonyl fluoride. The protein concentrations were measured using a BCA protein assay. Subsequent to being boiled in 100°C water for 5 min, equal amounts of total protein were separated by 8% SDS-PAGE. The protein was then transferred to polyvinylidene fluoride membranes (Millipore, Billerica, MA, USA). The membranes were blocked with 5% evaporated skimmed milk containing 0.1% Tween 20 for 2 h and then incubated with primary antibodies at 4°C overnight. The membranes were subsequently washed with Tris-buffered saline with Tween 20 (TBST) four times, followed by incubation with horseradish peroxidase-conjugated secondary antibodies for 1–2 h at room temperature. Following washing with TBST, the target protein bands were detected using an enhanced chemiluminescence detection kit with a ChemiDOC™ MP imaging system (Bio-Rad, Hercules, CA, USA).

### qPCR

Total RNA was extracted from the cells at the end of culture using TRIzol^®^ (Invitrogen Life Technologies). The primers of Fkn and GAPDH were designed by Beijing Sunbiotech Co., Ltd. (Beijing, China). The reverse transcription reaction was performed with 5 μg total RNA. For PCR amplification, cDNAs were amplified using SYBR Green qPCR Mix (Takara, Dalian, China); PCR protocols are summarized in Table I. The amplification reaction for each sample was performed in triplicate. Results are expressed as the ratio of Fkn to GAPDH mRNA.

### Statistical analysis

SPSS 16.0 (SPSS Inc., Chicago, IL, USA) was used for the statistical analysis. All data are expressed as the mean ± standard deviation and were analyzed using the Student’s t-test for two group comparisons or analysis of variance followed by the Student-Newman-Keuls test for multiple groups. P<0.05 was considered significant.

## Results

### AngII promotes RASMC proliferation and Fkn gene expression

**Figure 1:**
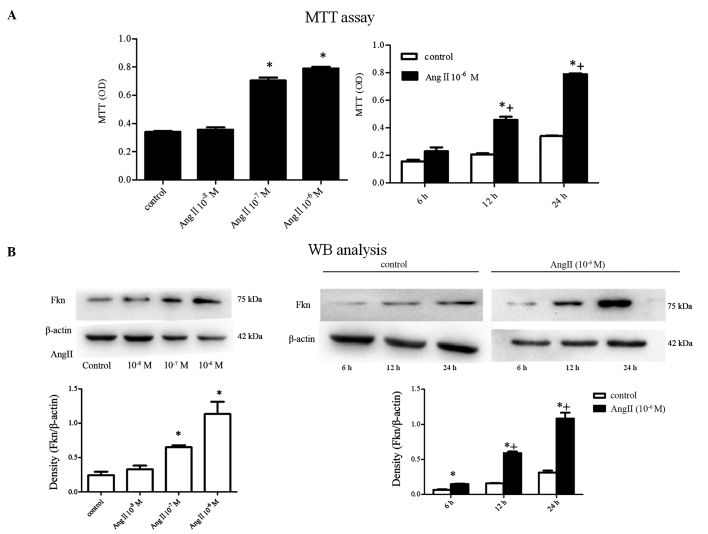
Proliferation of RASMCs induced by AngII and the expression of Fkn in response to AngII stimulation. (A) MTT assay showed that AngII induces RASMC proliferation in a time- and concentration-dependent manner. (B) WB analysis demonstrated that AngII also upregulates Fkn expression in a time- and concentration-dependent manner. ^*^P<0.05 vs. control; ^+^P<0.05 vs. AngII (1×10^−6^ M, 6 h). RASMCs, rat aortic smooth muscle cells; AngII, angiotensin II; Fkn, fractalkine; OD, optical density; WB, western blot.

MTT assay showed that AngII could promote RASMC proliferation in a time- and concentration-dependent manner (P<0.05; Fig. 1A). Accompanied by the enhanced proliferation of RASMCs, Fkn protein expression, as shown by western blot analysis, was significantly upregulated following the use of AngII, and the overexpression was also in a concentration- and time-dependent manner (P<0.05; Fig. 1B). By contrast, control cells only showed a baseline Fkn expression.

### Fkn gene silencing suppresses AngII-induced RASMC proliferation

**Figure 2:**
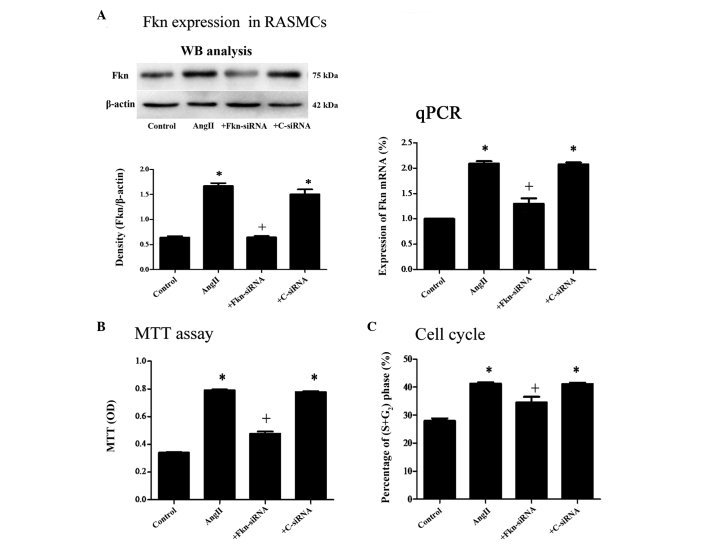
Effect of Fkn gene-knockdown on AngII (1×10^−6^ M)-induced proliferation of RASMCs. (A) Fkn siRNA inhibited Fkn expression at both mRNA and protein levels in RASMCs stimulated by AngII, while control siRNA showed no effects on AngII-treated RASMCs. (B) MTT assay and (C) Cell cycle analysis by flow cytometry showed that Fkn-knockdown also attenuated the AngII-induced cell proliferation. ^*^P<0.05 vs. control; ^+^P<0.05 vs. AngII. Fkn, fractalkine; RASMCs, rat aortic smooth muscle cells; WB, western blot; AngII, angiotensin II; qPCR, quantitative polymerase chain reaction; OD, optical density; siRNA, small interfering RNA.

Following Fkn siRNA transfection, the Fkn expression at the mRNA and protein levels in the AngII-stimulated Fkn siRNA group was suppressed in comparison with that in the AngII-stimulated control siRNA group (P<0.05; Fig. 2A). Furthermore, MTT assay and flow cytometry demonstrated that the Fkn siRNA transfection significantly attenuated the AngII-induced cell proliferation (P<0.05; Fig. 2B and C).

### Neferine inhibits the proliferation of both normal and AngII-stimulated RASMCs

**Figure 3:**
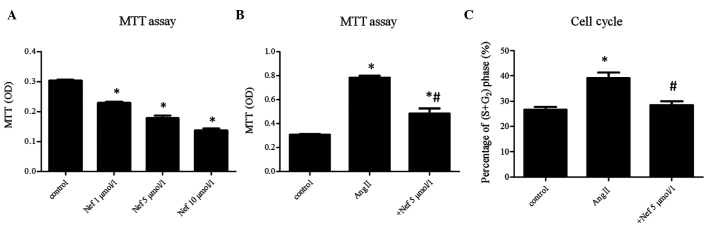
Anti-proliferative effect of neferine on normal and AngII (1×10^−6^ M)-stimulated RASMCs. (A) MTT assay showed a concentration-dependent anti-proliferative effect of neferine on normal RASMCs. (B and C) The anti-proliferative effect of neferine on the AngII-treated RASMCs was confirmed by (B) MTT assay and (C) cell cycle analysis. ^*^P<0.05 vs. control; ^#^P<0.05 vs. AngII (1×10^−6^ M). OD, optical density; AngII, angiotensin II; RASMCs, rat aortic smooth muscle cells; Nef, neferine.

MTT assay showed that neferine inhibited RASMC proliferation in a concentration-dependent manner in comparison with the control group (P<0.05). Neferine treatment at a concentration of 5 μmol/l appeared to result in moderate inhibition, and this concentration of neferine was used in the following experiments (Fig. 3A). MTT assay also showed that 5 μmol/l neferine attenuated the proliferative effect of AngII at 1×10^−6^ M (neferine pretreatment for 1 h followed by AngII treatment) (P<0.05; Fig. 3B). This inhibitive effect of neferine on the AngII-induced proliferation of RASMCs was further confirmed by cell cycle analysis using flow cytometry (P<0.05; Fig. 3C).

### Neferine attenuates AngII-induced Fkn expression in RASMCs

**Figure 4:**
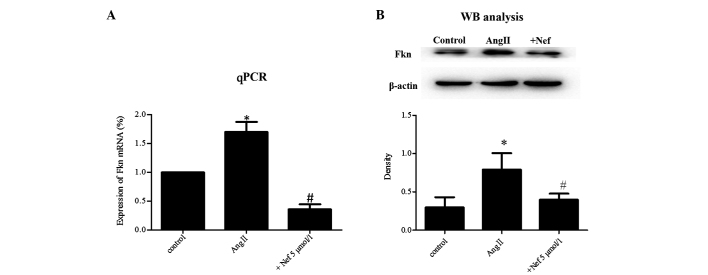
Neferine attenuates the AngII (1×10^−6^ M)-induced Fkn overexpression in RASMCs. (A) qPCR showed the mRNA expression levels under different experimental conditions; while control cells showed a baseline Fkn expression, AngII caused a significant overexpression of the Fkn gene. With neferine pretreatment, the overexpression of Fkn was greatly attenuated. (B) WB analysis showed a similar change in Fkn expression at the protein level among the experimental groups. ^*^P<0.05 vs. control; ^#^P<0.05 vs. AngII. qPCR, quantitative polymerase chain reaction; AngII, angiotensin II; Nef, neferine; WB, western blot; Fkn, fractalkine; RASMCs, rat aortic smooth muscle cells.

Fkn mRNA and protein expression in normal RASMCs was limited, but, following exposure to AngII for 24 h, Fkn expression was significantly upregulated (P<0.05). By contrast, pretreatment with neferine (5 μmol/l) markedly attenuated the enhanced Fkn expression at the mRNA and protein levels (P<0.05; Fig. 4A and B).

## Discussion

In the present study, the following observations were noted: i) AngII stimulated Fkn expression in RASMCs; ii) Fkn gene-knockdown attenuated the AngII-induced proliferation of RASMCs and iii) neferine inhibited AngII-induced Fkn expression, attenuating the proliferative effect of AngII on RASMCs.

Accumulating evidence has shown that Fkn plays an important role in cardiovascular diseases (12,25,26). Fkn is an atypical chemokine that exists in either a membrane-bound form or as a soluble chemokine (9). The membrane-bound form acts as an adhesion molecule and enables leucocyte adhesion via binding to chemokine (C-X3-C motif) receptor 1 (CX3CR1), while the soluble form functions as a chemoattractant for monocytes and T cells (27). Fkn is expressed in atherosclerotic plaques (28,29), and an absence of Fkn or its receptor, CX3CR1, prevents atherosclerotic lesion formation (30,31).

The proliferation of SMCs is involved in atherosclerosis and hypertension, and AngII is an important stimulus among the numerous involved (7,32). AngII contributes to the vascular SMC growth, endothelial dysfunction and vascular inflammation in hypertension. There is little Fkn expression in endothelial cells or SMCs under normal conditions; however, overexpression of Fkn has been observed when cells are under a certain stimulation, such as interferon-γ and tumor necrosis factor-α (9,23). Although there has been a report of AngII inducing Fkn overexpression in endothelial cells (16), the effect of AngII on Fkn expression in SMCs has not been documented to date. In this study, it was revealed that AngII upregulated Fkn expression in a time- and concentration-dependent manner, and knockdown of Fkn caused a significant decrease in cell proliferation in AngII-stimulated RASMCs.

In this study, neferine was again found to be capable of inhibiting AngII-induced RASMC proliferation, which was consistent with a previous study (22), and the anti-proliferative effect of neferine on AngII-stimulated RASMCs was revealed to be the result of suppressed Fkn expression. Neferine markedly attenuated the proliferative effect of AngII on the RASMCs by suppressing Fkn gene expression.

In conclusion, the findings in this study show that the Fkn gene plays an important role in the AngII-induced proliferation of RASMCs. Through Fkn, AngII exerts its proliferative effect on the cells, and by inhibiting Fkn expression, neferine exerts its anti-proliferative effects on the AngII-stimulated cells. Further studies are, however, required to fully understand the mechanisms and underlying signaling pathways of SMC proliferation.

## Figures and Tables

**Table I:** PCR primer sequences and protocol.

Gene	Primer sequences	Length (bp)	PCR protocol
Fkn	5′-ctcgtcccagagtgaggaag-3′ (sense) 5′-ctgctcctcaggcctacaac-3′ (antisense)	106	95°C/15 sec, 60°C/60 sec, 45 cycles
GAPDH	5′-agacagccgcatcttcttgt-3′ (sense) 5′-tgatggcaacaatgtccact-3′ (antisense)	142	95°C/15 sec, 60°C/60 sec, 45 cycles

PCR, polymerase chain reaction; bp, base pairs; Fkn, fractalkine.
